# Ten simple rules for funding scientific open source software

**DOI:** 10.1371/journal.pcbi.1010627

**Published:** 2022-11-17

**Authors:** Carly Strasser, Kate Hertweck, Josh Greenberg, Dario Taraborelli, Elizabeth Vu

**Affiliations:** 1 Chan Zuckerberg Initiative, Redwood City, California, United States of America; 2 Alfred P. Sloan Foundation, New York, New York, United States of America; Dassault Systemes BIOVIA, UNITED STATES

## Abstract

Scientific research increasingly relies on open source software (OSS). Funding OSS development requires intentional focus on issues of scholarly credit, unique forms of labor, maintenance, governance, and inclusive community-building. Such issues cut across different scientific disciplines that make them of interest to a variety of funders and institutions but may present challenges in understanding generalized needs. Here we present 10 simple rules for investing in scientific OSS and the teams who build and maintain it.

## Author summary

As funders of scientific research, we have a unique knowledge of the challenges that researchers face in finding support for the many parts of their projects. Scientific software is a central part of the research process and is increasingly in need of investments to ensure continued advancement and progress. Here we draw on our experiences as funders to provide guidance and considerations for funders and other community members interested in supporting scientific software. We address specific issues related to software, including contributor community development, governance, sustainability, and diversity and inclusion.

## Introduction

Virtually all of research now relies on computational tools and infrastructure. In many fields, open source software (OSS) represents the foundation of these computational capabilities. This is particularly true in data-intensive scientific disciplines: Given the proliferation of large datasets that require domain-specific analyses and complex infrastructure to process, the role of OSS has evolved to critical infrastructure for research.

Here we are defining scientific OSS as the source code, algorithms, scripts, computational workflows, and executables that are produced with the explicit intention of being used by others [1]. Unlike the majority of software and computational tools used in other areas, scientific OSS is most often produced by academic researchers and developed by globally distributed communities of scientists and contributors, ranging from students to professionals; this is in contrast to other open source projects that may be supported primarily by software engineers working at commercial/for-profit companies. For the purposes of this paper, we will focus on the particular subset of OSS intentionally developed for research and will refer to this scientifically oriented software as either OSS or simply software.

Despite the critical importance of OSS [2], its maintenance, continued growth, and improvement historically has been deprioritized by institutions, publishers, and funders as a less important byproduct of the research enterprise [3,4]. Institutions rarely give credit to researchers who invest time and energy in OSS [5], and funders often focus on novel development of new OSS rather than core support for existing and highly utilized OSS projects (e.g., [6–8]). While there is a body of research on the general practice of OSS [9–11], very little of that work speaks directly to the institutional context of science and much more research is needed.

The authors are funders who believe that investing in OSS is required to support the research enterprise. Several funding programs have emerged to support research software (and scientific open source projects in particular) at different stages of its lifecycle, both in the philanthropic and in the public sector. For an overview and analysis of the funder landscape, see this recent report by the Research Software Alliance [1]. Funding, however, is only one mechanism for investing in OSS. Other equally important ways of investing include making OSS a fundable and recognizable research output, explicitly designing programs tailored to the needs of scientific OSS communities, and recognizing the role of individuals and teams in supporting OSS projects. This is not necessarily a novel idea; many have argued that government funds [12,14], institutions [15], and publishers [16] should recognize and reward software development. Here we offer 10 rules for how to thoughtfully invest in OSS based on our experiences in private philanthropic organizations.

**Fig 1:**
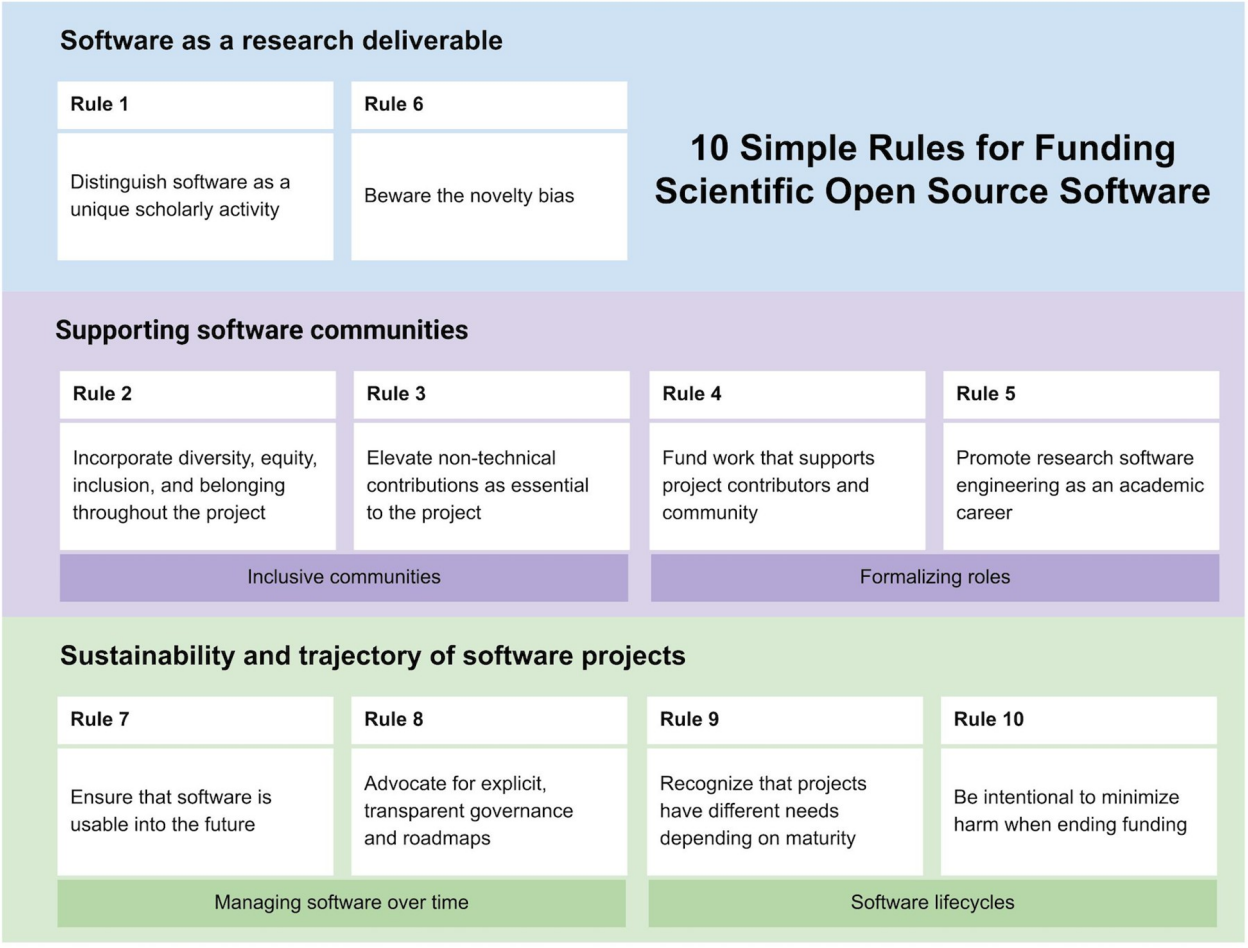
Ten simple rules for funding open source scientific software, grouped by themes. Numbers reference the order in which rules occur in the narrative.

The audience for these rules is primarily groups that invest in research. This includes traditional funders (federal, philanthropic, and other contexts), who will find utility in the rules below for landscaping grant opportunities, framing grant solicitations, and assessing both applications and funded projects. Fiscal sponsors can use these rules to maximize the potential of OSS projects in applying funds effectively. Another important audience includes academic institutions and other stakeholders that play a part in research incentive structures. If others in your organization are hesitant to invest in OSS, these rules may help you make a case internally for supporting software development and the teams performing this work. An additional audience for these rules is OSS developers and maintainers, as the rules provide guidance for ensuring a project is ready for investment. The final audience includes all researchers who use OSS (arguably, anyone currently performing scientific research). As individuals who review grant applications, manuscripts, and promotion and tenure materials, all scientists have the ability to advocate for OSS work as important, meaningful, and worthy of acknowledgement. These rules will resonate differently with each of these audiences. As presented in the narrative below, the rules are primarily organized to suit the mindset of funders interested in supporting OSS, ordered roughly chronologically according to the point at which a funder should consider each concept. An alternative interpretation of rule organization is presented in Fig 1; this structural and conceptual representation may be more appealing for other audiences.

With these 10 rules, we stand on the shoulders of giants. Groups that blazed the trail in this space—we consider them friends, peers, and fellow travelers—include the Research Software Alliance [17], the Software Sustainability Institute [18], the US Research Software Sustainability Institute [19], the Research Data Alliance [20] and FORCE11 [21] Software Citation Groups, the Software Heritage Foundation [22], and many others. We credit many of the ideas in this manuscript to discussions among groups in recent years.

### Rules

#### Rule 1: Distinguish software as a unique scholarly activity

The most fundamental rule for designing effective funding schemes in support of OSS is to treat software as a unique [23] and important [24] scholarly output. Well-intentioned programs aiming to support software development often borrow funding objectives, impact statements, and selection criteria from programs designed to fund *research*. This forces applicants to describe the impact of their software using language, narratives, and metrics that are not centered on software as a legitimate output and distort the unique contribution [25] software makes to science and scholarship.

Centering a program on software requires asking questions in the application process that directly relate to software adoption (e.g., providing space to talk about the impact of their work in a narrative format) and removing questions from the evaluation criteria that primarily assess impact through the lens of journal articles, citations, or other traditional measures of scholarly outputs. Many types of metrics exist that can help quantify the impact and maturity of a software project. For example, in programs run by our two organizations, we ask applicants to provide information about the existence of assets like developer documentation, contribution guidelines, end-user documentation, bug trackers, continuous integration systems, or a code of conduct. In addition, publicly available data from the code repository where a software project is hosted can also provide valuable insights into the volume of contributions, unique number of contributors, level of activity, forks, and dependencies. These types of indicators help provide a data-driven representation of the impact and maturity of a software tool as guidance to funders and reviewers (see Rule 9).

Shifting the perception of software as a legitimate scholarly activity also requires institutions and other evaluators to evolve the systems used to assess the impact and productivity of academic staff to include all research outputs, rather than focus on publications in prestige journals. Encourage and promote the adoption of software citation best practices [26] that make it easier for maintainers of OSS to track and surface the impact of their work, and consider funding for roles that lead the development and maintenance of OSS as a recognized and funded type of scholarly activity [27] (see Rules 3 and 5).

#### Rule 2: Incorporate diversity, equity, inclusion, and belonging throughout the project

While OSS aspires to democratize access and reuse of computational methods by all scientists, it is widely known that open source is predominantly created and maintained by a narrowly defined demographic. Open source maintainers are cisgender white men [28], and this pattern carries over to most open source projects widely used in science. This is particularly problematic since these projects often have loosely organized governance, and those shaping the project’s roadmap and priorities by default also determine who the projects serve. Lack of representation perpetuates the lack of diversity.

While racial and gender biases are frequently cited as problems in software development, diversity concerns include a much broader collection of personal identities and life experiences, including sexuality, disability, and expanded categorization for gender and race/ethnicity [29]. Improving diversity in software teams leads to better software, as a growing body of evidence supports that diverse teams increase novelty and impact of scientific outputs (e.g., [30]). In the context of software, prioritizing diversity, equity, and inclusion (DEI) in open source projects can help build communities that more accurately represent the people who will ultimately benefit from the work and use the software. Having contributions from people of all backgrounds—particularly those who are often underrepresented in OSS—can also support maintainers as they identify issues in their projects or communities, such as inherent bias in the software documentation or project roadmap [31]. Moreover, expanding the makeup and diversity of the teams that build these tools is critical to ensure that they can serve everyone and will inspire the next generation of scientists and developers. Ideally, approaches to ensuring diverse representation in a project, as well as inclusive practices, would be a part of the project goals from its inception. Unfortunately, DEI issues are often an afterthought, unintentionally sidelined until a project is well underway. This is particularly true for mature open source projects that began in an era where there was less emphasis on DEI in open source. Considering diverse participation as a secondary goal trivializes the work of underrepresented individuals and prevents the project from benefiting from diversity of thought throughout the software development cycle.

Encourage grantees and projects to think about DEI throughout the project. Specific goals and measures toward progress on DEI can ensure that such efforts are not waylaid by more technical project goals. For both CZI’s EOSS program and Sloan’s programs, a DEI statement is required that must be submitted alongside any proposal. These proposals are evaluated in part based on the statement, and we encourage incorporating metrics to evaluate progress. Rather than requiring that project maintainers learn how to implement DEI best practices themselves, encourage them to partner with organizations that have experience in this space. Fiscal sponsors like Code for Science & Society and Open Source Collective have begun working hand in hand with the OSS projects they support to encourage recruitment and retention of more diverse contributors. Groups like Outreachy, Data Umbrella, and PyLadies are all great examples of communities of practice that specialize in promoting and supporting underrepresented groups. Local communities of practice are also important: They have cultural, geographic, and human language expertise that can make open source interactions more welcoming for new participants from the area. Communities like CABANA [32] in Latin America and H3ABioNet [33] in Africa can not only support training activities, but also promote resilience for individuals contributing to projects hosted in underrepresented countries.

Ideally, considerations about DEI should be integrated throughout the grant making process, from initial ideation for a grant opportunity through award selection and reporting requirements. Such integration may represent a substantial shift in mindset from current processes, though, so it may be necessary to center DEI activities in more explicit ways to help promote this transition. One example is to support early-career scientists specifically from underrepresented groups who contribute to or lead OSS projects, as this may provide a crucial incentive to continue involvement in open source as they progress in career stage from trainee to long-term roles. Another option is to consider earmarking support for training, workshops, and access to resources for grantee cohorts on topics related to DEI, such as code of conduct training, accessibility, human language translation, and best hiring practices. These provide grantees with valuable resources they can apply to their projects, while also specifically targeting the development of DEI-centered values necessary for long-term strategy integration.

#### Rule 3: Elevate nontechnical contributions as essential to the project

It’s tempting to focus software funding only on development of new code, specifically for feature enhancements, which reflect other traditional scientific gains. However, one special aspect of building software is the collaborative, distributed work structure, with participating individuals possessing different skills and engaging in separate tasks. This rule encourages funders to acknowledge and support the myriad activities required for OSS to grow sustainably, and to call out this funding when setting up grants with project leads.

Traditional roles in which participants may engage in OSS range from software developer, requiring highly technical coding expertise, to users/researchers, who provide feedback about the utility of the software for their own research data and questions. Users with sufficient technical knowledge may also share bug reports and minor fixes. Contributions to the project that directly interface with software development, however, tend to disproportionately receive attention, acknowledgement, and funding.

Acknowledge contributions that are not focused on writing code, like improving software design, writing documentation, developing tutorials, advocating for the project, and formal testing of new product features (see Rule 4). In practice, this means that OSS projects should be encouraged to advertise structured ways that community members can participate, and provide acknowledgement for contributions that may not include additions to the code base.

From a funding perspective, project goals including nontechnical contributions should be valued similarly to code development and upkeep, and when assessing grant-funded work, project deliverables resulting from funding can and should include the results of different types of contributions. This line of reasoning also resonates with Rule 1: If we value software as a special type of scholarly activity, then multiple types of contributions to OSS matter in the same ways that different roles (laboratory scientist, data analyst, writer) matter to a research project. Moreover, if we elevate the importance of noncoding roles, we are also highlighting the contributions from roles that are traditionally gendered and/or held by members of otherwise underrepresented groups. As with Rule 2, however, it’s important to remember that these efforts represent a fundamental shift in how work supporting projects and communities is valued. The intention is not to expect only these types of contributions from underrepresented groups, but instead, such that it becomes more of a shared responsibility.

#### Rule 4. Fund work that supports project contributors and community

Having established that diverse roles matter in the context of an OSS project (Rule 3), scalability has the potential to become an issue: More roles (and more diverse participants) require additional support to connect parts of the project. We propose that the solution to this dilemma is to fund staff that support contributors and the broader community, in addition to the primary owners/maintainers [34]. Many OSS leaders began as scientists who made a second career of coding. A mature OSS project will eventually reach a point at which it is impossible for a single person to adequately manage the code development, apply the code to the scientific questions at hand, and manage the personnel and strategy of the overall project. Specifically, funding additional roles related to project and community management is an important part of sustainably growing an OSS project.

OSS is only relevant because of the community surrounding it, and that community requires investments of time, energy, and, ideally, money to properly develop and thrive. Community managers, sometimes also called community advocates, work externally to the core development team to encourage dialogue with contributors and users (e.g., the work done by CSCCE; [35]). Similarly, project managers work alongside the core staff to help organize, prioritize, triage, and delegate tasks, not just related to code development, but also synthesizing community feedback into actionable tasks. Project management can cover a wide array of tasks, from onboarding personnel to integrating community governance, and is essential for ensuring the core team can work effectively together and with the broader community [36]. This differs from *product* management, which is more technical in nature and focuses on user stories, use cases, and software requirements, and tends to already be a focus of work for software developers.

Funders can encourage a robust and welcoming community by supporting capacity building as a critical component of an OSS project’s life cycle. Grantees may not even be aware that funding is possible for such positions, but when properly integrated with the software development process, these roles can yield significant gains to the project as a whole. By providing opportunities for growth and learning, and ensuring coordination among various aspects of the project, the community can expand in the longer term to ensure sustainability [10]. The goal for this type of funding is acknowledging and increasing visibility of the roles that the project most needs at any given time, which ultimately amplifies the work of software developers.

#### Rule 5: Promote research software engineering as an academic career

As research teams are becoming increasingly dependent on software to do their work, new types of roles within those teams have risen in profile. In particular, the Research Software Engineer (RSE) has become a more recognized professional role focused on combining software engineering expertise with experience in specific research disciplines. These roles are seen as valued methodological collaborators to researchers across disciplines, bringing value to specific research projects by enabling the software ecosystem needed to spur scientific advances. With a specific role focused on building robust software, the durability of methods and findings produced by research labs is no longer dependent on the capabilities and timelines of other less stable sources of research software laborers, i.e., the rotating graduate student or overstretched principal investigator.

RSEs represent a new role for academia, requiring new job codes, updated pay scales, and changes to long-standing hiring and promotion practices. Further, there are tensions with RSE work and the incentives in academic environments. RSEs may see themselves as researchers with software skills that don’t serve them well as someone working toward tenure or permanent contract. Alternatively, they may choose to engage in software engineering full time within their research labs and thereby choose to spend time on software that isn’t yet considered a first-order research output (see Rule 1). As funders invested in creating healthy research ecosystems, it is important that we give talented individuals the opportunity to build their skills as an RSE, should they decide they want to take it, with the assurance that their career isn’t completely invalidated. Programs centered on software development as a primary activity and targeted at early career contributors, in particular, are important tools for seeding and recognizing impactful work in support of longer-term careers in OSS.

A number of institutions and groups exist that recognize and elevate the importance of an RSE role in academia. Some examples include the Society of RSE in the UK [37], US-RSE [38], and other national RSE associations [39]. These groups organize a number of activities in support of and driven by RSEs in academia such as fellowship programs, training opportunities, and advocacy efforts. Funders can not only support these efforts directly, but also encourage equal leadership representation of lead RSEs as principal investigators on grant applications with significant OSS components.

#### Rule 6: Beware the novelty bias

Novelty is a desirable goal for traditional discovery-oriented research funding, but a less useful metric when funding OSS. As Andreas Mueller, a core developer on the scikit-learn project, noted: Funding programs designed to support “projects that do not exist yet… or to extend projects that are developed and used within a single research lab” are at odds with the reality of OSS [40]. By and large, successful open source projects that underpin the computational needs of science are built and maintained by communities and a large collective of contributors. Contributors to OSS, particularly scientific OSS, are a limited resource (see Rule 5). Funding schemes that support new software where credible and well-established alternatives already exist at best create new projects in an already crowded and unsustainable space [41], and, at worst, fragment an existing and limited pool of potential contributors.

Funders have the power to direct the attention of the broader community toward critical activities that may otherwise be neglected or overlooked. In the context of OSS, investments should therefore focus on directing new contributors’ attention and resources to projects that are mature, largely adopted, and have implemented robust governance and community management practices (see Rules 6, 7, and 8). We are not suggesting that there is no need to ever fund new projects; supporting new research and computational methods will always be important. However, the novelty bias has the potential to result in a million flowers blooming, and almost as many dying.

Funders can ask for information and metrics at the proposal stage to ensure that proposers make a good case for new funding or otherwise identify existing software upon which they can build their work or that existing projects are working toward robustness and sustainability. Examples of such metrics may include number of contributors; age of repository; availability of code, documentation, and contributor guidelines; the presence of a code of conduct; and dependencies. Many funders ask both the principal investigator and external peer reviewers about existing research in the area of the proposal; however, consider explicitly requesting a landscape analysis describing how the applicants’ OSS project stacks against similar software tools, or leverages existing tools in their novel approach. Similarly, metrics for OSS can help identify a project’s stage (see Rule 9); evidence of adoption and usage, both quantitative and qualitative, can help assess if a research software project is the de facto standard in the field or one among many.

#### Rule 7: Ensure that software is usable into the future

Funders want to maximize the value created by their investments, including OSS projects. While nothing is a guarantee, there are a number of ways to set up projects for long-term viability. This is particularly important for software that could be considered infrastructure—i.e., software that is depended upon by other software projects rather than those directly developing it.

With regard to technical choices, encourage OSS maintainers to build on existing technical successes, adding to the ecosystem rather than creating a new one. One of the greatest dangers to the sustainability of an OSS project is loss of engagement, and every idiosyncrasy can narrow the number of future users as well as contributors. Grantees should have clear justifications for deviations from mainstream languages, libraries, and technologies. Beyond technical details, the number of future contributors to a project can be heavily dependent on the clarity of its governance, and, while different models are appropriate to different projects [42,43], funders should at a minimum ensure that project governance is clearly articulated.

In order to be used, software needs to be maximally accessible and open for the long term. Open source licenses [44] can lock in openness, though they come with trade-offs (viral licenses are more robust in those protections, but less restrictive licenses may enable greater use). Beyond which license is chosen, it’s important that the code itself remains accessible; for guidance, one can look to recent efforts to establish FAIR principles for research software [13]. Projects should maintain an active code repository (ideally on a mainstream platform) and also commit to a plan for longer-term archiving and stewardship. Good documentation practices can also lower barriers for both users and contributors and should be an activity directly funded for both new and existing OSS projects.

Following a popular analogy, investing in an OSS project can be like adopting a “free puppy [45,46], with the ongoing costs of care and feeding far surpassing any initial development costs. It is likely that funders will want to transition projects to other sources of support for maintenance costs; these can include direct institutional or consortial funding, or cross-subsidies in the form of volunteer contributors.

Every software project is rife with trade-offs between the present and the future, and it’s important for funders to encourage frank discussion in proposals and reports of when technical debt is accrued and when it is paid down [47]. Software is subject to a form of entropy, drifting out of sync with dependencies and technology stacks over time, and funders should encourage and fund continued attention to business and/or community models that will support ongoing maintenance. At a minimum, a good software management plan can guarantee long-term access by relying on source code archives like Software Heritage [22], as well as platforms like Emulation-as-a-Service Infrastructure [48] to ensure executability into the future.

#### Rule 8: Advocate for explicit, transparent governance and roadmaps

OSS developed in the process of research has the potential to be less sustainable than other types of OSS as a direct result of its origins: Often these tools are developed to solve a specific problem or answer a nuanced question that a researcher encounters. Because they are designed as a means to an end, OSS projects developed for research (especially relatively immature projects) are more susceptible to poor documentation, a lack of transparent governance, and a lack of planning for the long term. Nonengineering-related elements of the OSS project—such as the adoption of user-centered design practices and a responsiveness to user needs—are likely to be neglected for work that contributes to obtaining research results (see Rule 3), especially since much of the incentives for academia are not aligned with best practices for software (see Rule 1).

Funders should consider these pitfalls when funding projects and ask questions at the beginning of the funding process to ensure that maintainers are thinking about sustainability and the long term (see Rule 7). This can be done formally by requesting a software management plan (e.g., [49]), or more informally by using a checklist to start conversations about governance and management (e.g., [50]). These approaches can ensure that the right decisions are made and a project is set up for long-term success and sustainability.

#### Rule 9: Recognize that projects have different needs depending on maturity

In supporting OSS projects, it is important to take into account their different stages of maturity. Projects may be at a prototype stage, in a growth phase, in maintenance mode, or simply be an idea sketched on paper. Assessing a project’s maturity and corresponding needs cannot be based on a simple set of metrics. A project’s audience size, for example, may or may not be a reliable indicator of the project’s maturity and future growth: Legacy projects may have accumulated a large number of forks or downloads while having completely ceased substantial development and regular maintenance; conversely, relatively new projects may have reached a significant level of maturity in terms of open source practices, community stability, and engineering practices that may not translate to usage and contributor metrics, particularly if they serve a particular niche in science. Similarly, qualitative data (collected through the application process) can help funders assess the maturity of specific open source practices that are hard to glean from project metrics: Evidence of a code of conduct in a repository, for example, may be considered as a minimum, binary indicator of a project’s commitment to principles of inclusion. A code of conduct tailored to the project’s specific community and with a clear process for responding to incident reports is a stronger indicator of how mature a project is in honoring its commitments. Understanding and appreciating these various stages, and relying on appropriate quantitative and qualitative data to assess them, will inform how to best support project owners and communities as they navigate the various stages of maturity.

Recognizing these different stages will also help identify where support is best aligned with a funder’s goals, and where other colleagues or organizations may potentially provide complementary support. For example, some funding programs may be more inclined to directly support the technical development of new software (and often it is easier to make a case for innovation-driven research; see Rule 6), whereas other programs may prioritize community engagement or maintenance. Relatedly, funder coalitions can play a unique role in supporting OSS by engaging in a community-informed consideration of needs through the early to late stages of a project, playing to the strengths and priorities of each philanthropic group.

Having a finger on the pulse of the different stages of OSS project maturity can also enable funders to help projects when unexpected changes or challenges arise. Software is not static, and a successful OSS project will evolve with user needs and as the broader context of the tool shifts. OSS projects may merge or fork depending on the needs of their communities, and funders should maintain awareness of the landscape of relevant projects in order to avoid duplication of effort or projects that drift out of sync with community priorities. Conversely, a real risk for OSS projects is more rapid adoption and scaling sooner than anticipated, resulting in something that has been called “catastrophic success” leading to “scalar debt” [51]. It is important to be cognizant of these possible scenarios and consider larger initial investments in promising projects to ensure that they are nurtured through successive stages of growth.

#### Rule 10: Be intentional to minimize harm when ending funding

Not all projects can or should be supported by philanthropic funding into perpetuity, so it is also valuable to understand what conditions should be in place as funders exit a funding relationship. No software project is ever actually finished; project maintainers simply run out of money, time, or interest. Grant funds can perpetually solve the first problem, but perhaps not the second or third, so be aware of “brittle projects that are overdependent on any one individual.” When deciding whether to continue active funding, pay attention to signals of demand from users, which can be a better indication of ongoing value and need for additional features than the views of the developers themselves.

That said, avoid the sunk cost fallacy—just because an OSS project has been funded in the past doesn’t mean that it should continue to be forever. In a context of finite grantmaking resources, you can only fund new things if you stop funding old ones. Funder “exits” should aim to minimize disruption and harm to the project, and many of the rules outlined above when well applied will set a project up for success beyond any one grant or funder. Funder coordination (for example, referring unfunded proposals to other, similar schemes) and funding coalitions (providing complementary types of support for different aspects of OSS maintenance and development) can help mitigate the impact of single-funder dependency and premature funder exits. A continued emphasis on sustainability, institutionalization, development of community and financial models, and integration with platforms, companies, and other projects can ensure the resilience of software projects as individual funders’ priorities and strategies change. And when it is time for active development on a project to end, long-term archiving strategies from source code to virtual machines and emulation mean that a dormant project can be resurrected on demand should the need arise.

## Conclusions

As datasets and computational power continue to grow, the role of OSS will also grow. Funders can help ensure that these projects are as useful, stable, and sustainable as possible by following the 10 rules above. A common thread in our rules is that support for OSS is not simply about funding a research project’s software development; instead, when supporting OSS funders should support people, not projects. The communities—and the people that compose them—are foundational to the projects (Fig 1). The roles required to accomplish work for a project go beyond a standard software engineer or a self-taught scientist who codes. Successfully running an impactful OSS project requires thoughtful community development, careful attention to DEI, and considerations for governance, management, and sustainability. Supporting OSS effectively and with an eye to the long term requires a holistic, people-centered approach.
